# ATM Alters the Otherwise Robust Chromatin Mobility at Sites of DNA Double-Strand Breaks (DSBs) in Human Cells

**DOI:** 10.1371/journal.pone.0092640

**Published:** 2014-03-20

**Authors:** Annabelle Becker, Marco Durante, Gisela Taucher-Scholz, Burkhard Jakob

**Affiliations:** 1 GSI, Helmholtzzentrum für Schwerionenforschung GmbH, Darmstadt, Germany; 2 Technical University of Darmstadt, Darmstadt, Germany; Laval University Cancer Research Centre, Canada

## Abstract

Ionizing radiation induces DNA double strand breaks (DSBs) which can lead to the formation of chromosome rearrangements through error prone repair. In mammalian cells the positional stability of chromatin contributes to the maintenance of genome integrity. DSBs exhibit only a small, submicron scale diffusive mobility, but a slight increase in the mobility of chromatin domains by the induction of DSBs might influence repair fidelity and the formation of translocations. The radiation-induced local DNA decondensation in the vicinity of DSBs is one factor potentially enhancing the mobility of DSB-containing chromatin domains. Therefore in this study we focus on the influence of different chromatin modifying proteins, known to be activated by the DNA damage response, on the mobility of DSBs. IRIF (ionizing radiation induced foci) in U2OS cells stably expressing 53BP1-GFP were used as a surrogate marker of DSBs. Low angle charged particle irradiation, known to trigger a pronounced DNA decondensation, was used for the defined induction of linear tracks of IRIF. Our results show that movement of IRIF is independent of the investigated chromatin modifying proteins like ACF1 or PARP1 and PARG. Also depletion of proteins that tether DNA strands like MRE11 and cohesin did not alter IRIF dynamics significantly. Inhibition of ATM, a key component of DNA damage response signaling, resulted in a pronounced confinement of DSB mobility, which might be attributed to a diminished radiation induced decondensation. This confinement following ATM inhibition was confirmed using X-rays, proving that this effect is not restricted to densely ionizing radiation. In conclusion, repair sites of DSBs exhibit a limited mobility on a small spatial scale that is mainly unaffected by depletion of single remodeling or DNA tethering proteins. However, it relies on functional ATM kinase which is considered to influence the chromatin structure after irradiation.

## Introduction

DNA double strand breaks (DSBs) arise from natural cellular processes as well as from external damaging agents like ionizing radiation and represent one of the most dangerous types of DNA lesions. Repair of DSBs is essential for cell survival and failure or misrepair can lead to genomic instability and the development of cancer through the generation of chromosomal rearrangements. The cellular response to DSBs is multifaceted and starts with a complex signal cascade to promote recruitment of DNA repair factors, chromatin alterations surrounding the break sites and cell cycle arrest [1], [2]. The organization of chromatin itself as well as its radiation-induced modifications influence repair processes in several ways and repair kinetics are strongly dependent on chromatin structure [3], [4].

Two mayor classical pathways of DNA repair are homologous recombination (HR) and non homologous end joining (NHEJ). In HR DSBs are repaired correctly by the use of the undamaged homologous sequence as a template, whereas NHEJ fuses broken DNA ends together, a process which can lead to chromosome exchanges especially if multiple breaks are present. In this case it is yet unclear what promotes the joining of DSB ends, but proximity and movement of the ends seem to play an important role [5]. In yeast persisting DSBs move to the nuclear periphery and form repair centers [6]–[8], which implies an aimed movement of individual DSBs. Moreover a higher mobility of damaged chromatin compared to non damaged chromatin was observed after induction of DSBs in yeast, most likely to facilitate homology search [9].

Early investigations in mammalian cells showed that chromatin exhibits mobility that can be described by a constrained random walk [10]. More recent work points to a relatively stable position of DSB containing chromatin domains [11]–[13], although a local expansion of chromatin after DNA damage was described [14], [15]. Movement analyses of individual breaks induced by restriction enzymes showed a dependency on Ku80 of the ability of break ends to locally diffuse [13]. An influence of repair proteins on chromatin mobility was also shown in a study on uncapped telomeres, which can be considered as one-ended DSBs and are processed accordingly [16]. The authors observed a reduction in mobility of uncapped telomeres in 53BP1 and ATM deficient cells [17]. Furthermore, the movement of damaged chromatin in mammalian cells seems to be enhanced by the induction of DSBs [18]. Mobility of DSBs could influence the frequency of chromosome rearrangements, a hallmark of carcinogenesis, especially if multiple DSBs are induced in close proximity by densely ionizing charged particles. At heterochromatic DSBs a distinct local decondensation of the surrounding chromatin was demonstrated directly after irradiation with charged particles [19], [20]. This local decondensation might be responsible for the described enhanced mobility of broken chromatin as suggested previously [14]. So far these radiation-induced chromatin dynamics are not well understood regarding their physiological consequences or proteins involved.

To gain more insight into this topic, we used a live cell approach to examine the influence of factors involved in chromatin decondensation, DNA repair or stabilization of damaged chromatin on the mobility of IRIF. In addition we took advantage of the high localized dose deposition of low energy charged particles where substantial decondensation is known to occur [19], [20]. Linear tracks of DSBs in 53BP1-EGFP expressing U2OS cells were generated by low angle irradiation [21] and followed up by live cell imaging. We found that chromatin mobility was not affected by most tested chromatin modifiers and DNA repair proteins on the measured temporal and spatial scale. However we show that DSB mobility strongly depends on ATP and functional ATM kinase.

## Materials and Methods

### Cell culture and irradiation

U2OS cells stably expressing 53BP1-GFP or NBS1-GFP cells (kindly provided by Dr. Claudia Lukas Danish Cancer Society, Copenhagen, Denmark), described previously [22]–[24], were grown in DMEM (Biochrome, Berlin, Germany) supplemented with 10% FCS and 1 μg/ml puromycin under standard conditions (monolayer cultures, 95% air, 5% CO_2_, 37°C, 100% humidity). For siRNA treatment and irradiation cells were seeded on round 30 mm glass coverslips. Charged particle irradiation was performed at the accelerator facility of the GSI Helmholtzzentrum für Schwerionenforschung using C (LET (linear energy transfer):170 keV/μm; dose: 0.54 Gy), Cr (2630 keV/μm; 8.4 Gy), Pb (13500 keV/μm; 43 Gy) or U (15000 keV/μm; 48 Gy) as indicated. Choice of ions used in the experiments was mainly determined by the availability of beamtime at the accelerator. There was no indication of a LET dependent change of the mobility for the used particles. In this study cells were irradiated at an angle of 15° as described [25] with a fluence of 2×10^6^ particles/cm^2^. For photon irradiation an X-ray tube (Isovolt Titan 320-13) operated at 250 kV and 16 mA (GE Sensing & Inspection Technologies GmbH, Ahrensburg, Germany) was used. The X-ray beam was additionally filtered by 1 mm Cu and 1 mm Al to remove low energy photons. Cells received a dose of 1 Gy X-rays.

### siRNA mediated knockdown, inhibition and western blot

Knockdowns were performed using the INTERFERin transfection kit (PeqLab, Erlangen, Germany) according to the standard protocol. SiRNA sequences can be found in tab. S1. Efficiency of each knockdown was analyzed by western blot 48 h after treatment following a standardized protocol [26] and quantified using ImageJ software. When applicable further knockdown verification was done by immunofluorescence assays. Antibodies used were: ACF1/BAZ1A (Bethyl Laboratories, Montgomery, USA), MRE11 ab214 (Abcam, Cambridge, UK), NIPBL (Santa Cruz, Heidelberg, Germany), PARG (Merck Millipore, Billerica, USA), PARP (Cell Signaling, Danvers, USA), SMC1 (Cell Signaling, Danver, USA). 10 mM caffeine (Sigma Aldrich, Hamburg, Germany) was used for unspecific inhibition of ATM and 15 μM KU55933 (Merck Millipore, Billerica, USA) for specific ATM kinase inhibition. PARP1 was inhibited by 10 μM PJ34 (Sigma Aldrich, Hamburg, Germany). Inhibition of all proteins was started two hours before irradiation and efficiency of inhibition was analyzed by immunofluorescence staining as described in the results. Depletion of ATP was carried out by 10 mM sodium azide and 50 mM 2-desoxyglucose diluted in culture medium 30 minutes prior to irradiation, resulting in partial depletion to ensure the formation of repair foci.

### Immunofluorescence assay

For the immunofluorescence staining of pATM cells were fixed at room temperature in 2% formaldehyde (15 minutes) and permeabilized in 0.5% triton X-100 (10 minutes). MRE11 staining is applied after extraction of soluble proteins as described previously [21]. For staining of poly-ADP-ribose (PAR) cells were fixed following a standardized protocol [27]. Staining of PAR (clone H10, Oncogene, San Diego, USA) and pATM (anti-phospho-(Ser1981)-ATM, Rockland, Gilbertsville, USA) was performed with a dilution of 1∶200 and 1∶750 for MRE11 ab214 (Abcam, Cambridge, UK) in 0.4% BSA in PBS for one hour at room temperature. Primary antibodies were detected with 5 μg/ml Alexa 488 or 568 goat anti-mouse IgG conjugate (Molecular Probes, Leiden, The Netherlands) (1 hour, room temperature) respectively and counterstained with 1 μg/ml DAPI (20 minutes, room temperature).

### Live cell microscopy and data analyses

Immediately after irradiation live cell observations were performed using a spinning disc confocal microscope (Nikon Eclipse Ti with Yokogawa CSU_X1) equipped with a temperature controlled chamber stably adjusted to 37°C and 5% CO_2_. Ten to twenty XY positions were selected and Z-stacks (5 to 15 slices at a distance of 0.3 or 0.5 μm) were recorded. Positions were revisited automatically at selected time intervals of 2 to 5 minutes over a time period of 1–2 hours starting about 20 minutes after irradiation. Excitation levels were kept below 7.5 μW in order to avoid photobleaching and cellular stress. Identification of foci was done in Huygens essential software (SVI, Hilversum, The Netherlands) basically by following them on a frame to frame basis in a deconvoluted and registered 4D dataset. The assignation is based on intensity and spatial information in consecutive time-frames. The intensity weighted center of a focus is than calculated and connected to a motional track. These estimated motional tracks where checked for inconsistencies by visual inspection. Only correctly identified tracks were used for the analysis. The mean square displacement (msd) from the track origin was averaged for all IRIF under the given conditions and was plotted against time for each experiment where n represents the number of nuclei analyzed. Several foci were scored per nucleus.

## Results

**Figure 1:**
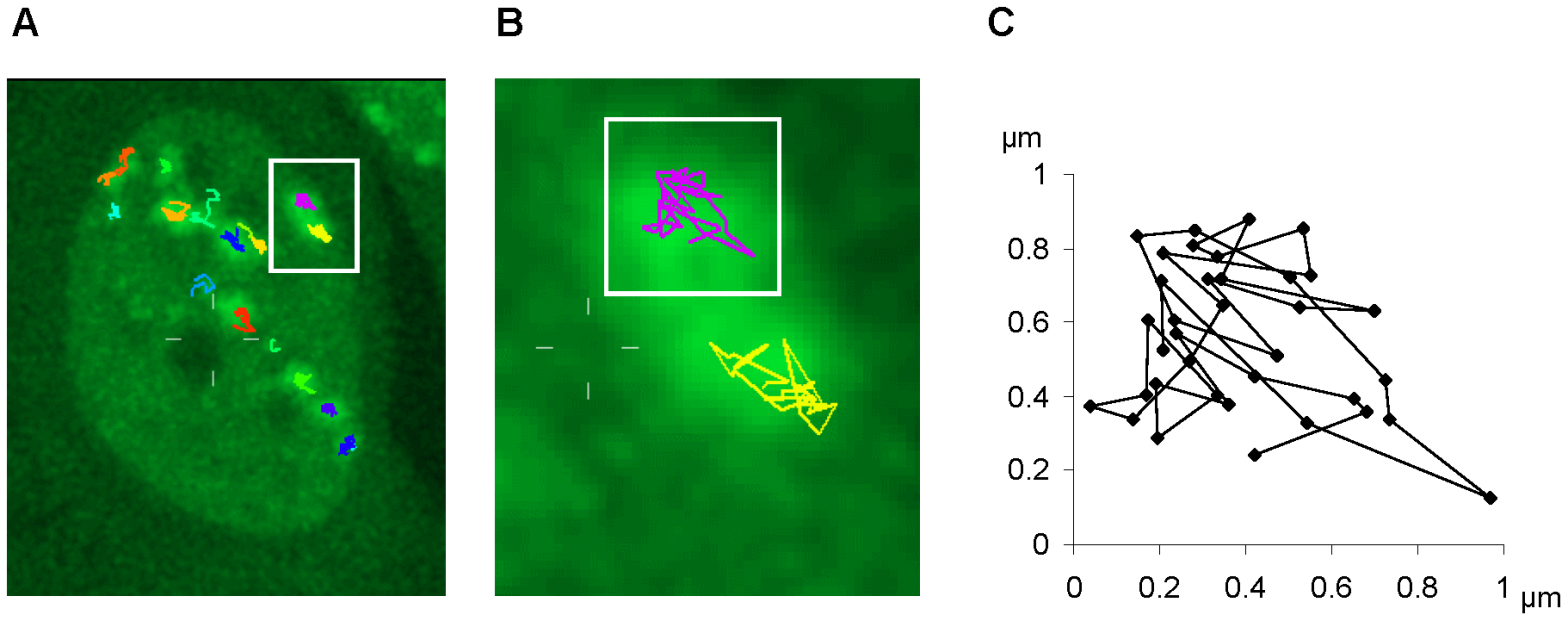
Mobility measurement of 53BP1 IRIF in U2OS-cells. A) 2D maximum projection of a U2OS cell nucleus after irradiation with Cr ions (LET: 2360 keV/μm) with 53BP1-GFP accumulation (green foci) along the ion trajectory at sites of DSBs. Colored tracks represent movement of 53BP1 foci measured by 2D tracking within three hours of observation. B) Magnification of two exemplary tracks of 53BP1 mobility indicated by the white square in A. C) Spatial mobility of the in B) selected 53BP1 track over three hours.

To investigate in a live cell approach if mobility of clustered DNA lesions is dependent on chromatin modifications we used heavy ion irradiation of human osteosarcoma cells (U2OS) stably expressing 53BP1-GFP. Irradiation with charged particles induces high numbers of DSBs in close proximity. In this case focal accumulations of repair proteins do not necessarily represent single DSBs as induced by sparsely ionizing photon radiation but rather clusters of many DSBs [21]. The quantity of DSBs generated by an ion traversal scales with the linear energy transfer (LET) and is around 3–4 DSBs/μm for carbon ions and in the range of several hundred DSBs/μm for the heavier ions used in this study [21], [28], [29], whereby one μm roughly corresponds to the size of one 53BP1 focus. 53BP1 is a key regulator of DSB repair which accumulates at sites of DSBs in distinct foci colocalizing with γH2AX and persisting during ongoing repair [30]. By tracking individual 53BP1 foci we confirmed a general positional stability of damaged chromatin domains with limited mobility in a submicron range (Fig 1).

A high percentage of radiation induced DSBs are repaired during the first hours following induction of DSBs both with X-rays [31] as well as with a wide range of charged particle irradiation [32]. Therefore, the first hours might be a critical time-window for the formation of chromosome rearrangements and accordingly, we performed our analyses during this time frame.

By analyzing the curvature of a msd plot, normal diffusion behavior can be distinguished from deviations like directed or anomalous diffusion or confined mobility. Normal diffusion results in a linear slope with a constant diffusion coefficient over time, whereas anomalous subdiffusion is commonly defined via a power-law dependent increase of the mean square displacement yielded by the equation



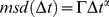
(eq1)with α<1; where α represents the anormality parameter and Г represents the transport coefficient [11]. Subdiffusion occurs in various biological systems and is caused by viscoelasticity of the surrounding medium, obstruction by immobile obstacles and binding events [33], [34]. Computer simulations of a measured subdiffusive motion of IRIF recently predicted an enhanced probability of rejoining correct ends of DSBs compared to normal diffusion [11]. In contrast to subdiffusion, confined motion is implicated into a constricted volume given by the confinement radius r_c_. A confinement can result from a restrictive volume, a trapping in a certain domain or by tethering to a nuclear structure [35] which leads to a bending of the msd curve. In the case of confined diffusion the msd is yielded by



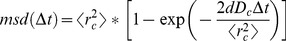
(eq2)with the diffusion coefficient D_c_ and the respective diffusion radius r_c_ [36].

Fitting our experimental data revealed that mobility of 53BP1 foci in irradiated U2OS cells can be described similarly well by either subdiffusional movement or confined diffusion. Generally, we obtained good correlations of both types of fits (R^2^>0.99) for wildtype cells.

**Figure 2:**
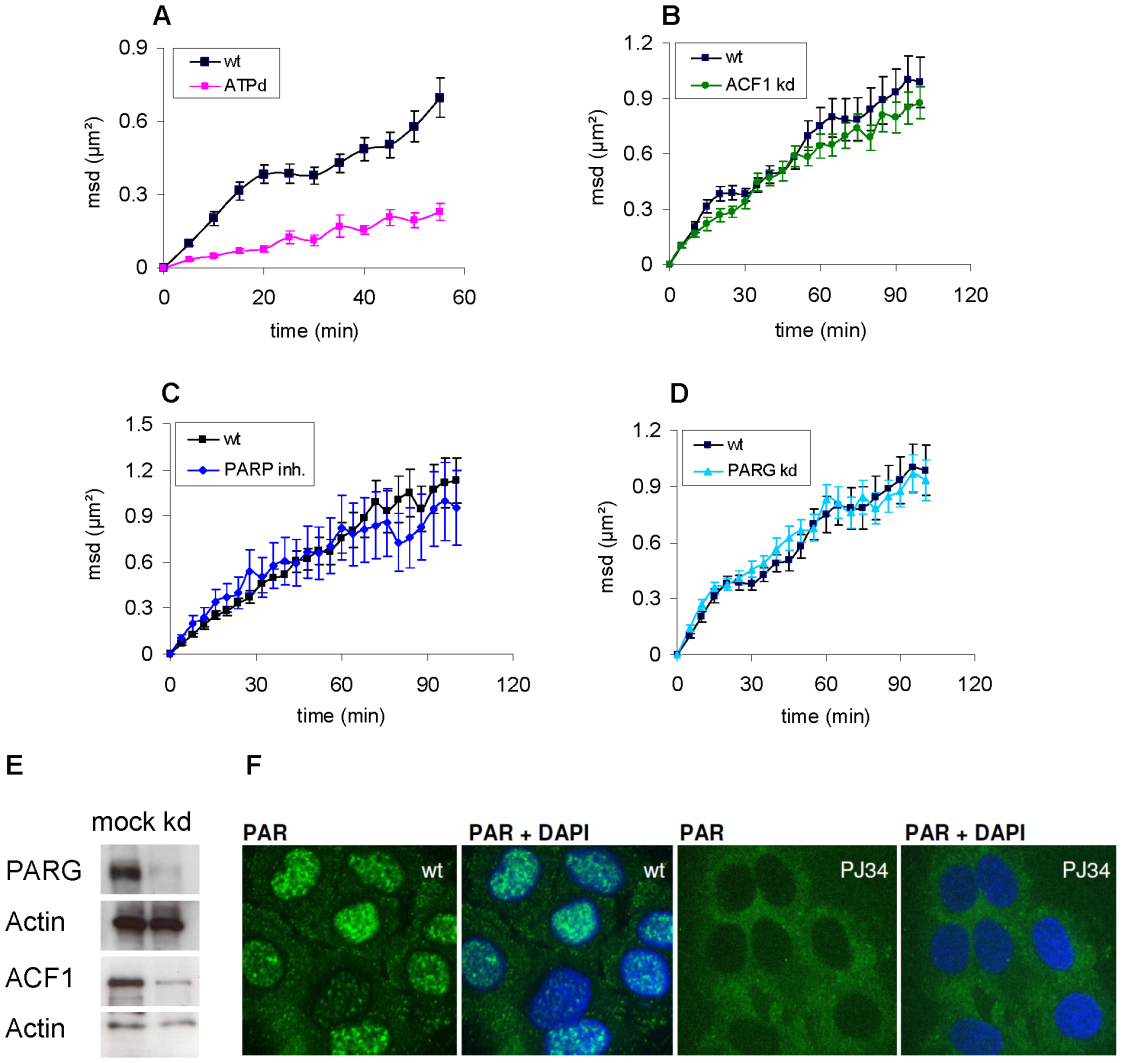
Influence of repair-related chromatin modifying proteins on mobility of 53BP1-GFP foci in irradiated U2OS cells. A) Plot of the mean square displacement (msd) of IRIF in control cells (wt) and cells which were depleted for ATP 30 min prior to carbon ion (LET: 170 keV/μm) irradiation (n = 7). Errors represent SEM in all plots. B) Msd of IRIF in cells after knockdown of ACF1 (n = 23) and non treated controls (wt) (n = 11) after irradiation with Cr (LET: 2360 keV/μm). C) Comparison of the msd of IRIF in cells after inhibition of PARP (10 μM PJ34) and controls (wt) (n = 15). Cells were irradiated with C (LET: 170 keV/μm). D) Msd of IRIF in cells after knockdown of PARG (n = 15) and in non treated controls (wt) (n = 11). Cells were irradiated with Cr (LET: 2360 keV/μm). E) Western Blot showing the ACF1 and PARG knockdown efficiency with actin as loading control. F) U2OS cells treated with 20 mM H_2_O_2_ for 10 min, fixed and stained for PAR (green) and DNA (blue) show efficiency of PARP1 inhibition with 10 μM PJ34.

Since energy dependent chromatin remodeling influences both the movement of undamaged chromatin [37] as well as the mobility of IRIF [18] we used ATP depletion [38] as a benchmark to validate our tracking system. For wildtype (wt) U2OS cells, we measured a msd of 0.7 μm^2^/h for the first 60 min similar to the one described previously using a different microscopic setup and analysis in 2D [28]. In contrary to the wt, cells which were depleted of ATP exhibited a drastic reduction (∼66% of msd after 1 h) of IRIF mobility (Fig. 2A). By depletion 30 min prior to irradiation a low level of ATP persisted during irradiation enabling the formation of repair foci. As cells were kept in depletion media during our measurements, ongoing energy starvation led to disintegration of repair foci that started around one hour after irradiation and restricted the observation time. The obtained results verify that metabolic perturbation can lead to pronounced differences in IRIF mobility which can be detected by our approach.

### The extent of DSB mobility is not influenced by polyADP-ribosylation or the chromatin remodeler ACF1

Chromatin remodelers rearrange, move or eject nucleosomes in an ATP-dependent manner. These alterations influence the chromatin structure and regulate packing of DNA during processes like replication, transcription or DNA repair. To determine if the dramatic reduction of IRIF mobility we observed after ATP depletion can be attributed to the absence of radiation-induced local remodeling, we knocked down ACF1, a chromatin remodeler of the ISWI family which is known to participate in DSB repair and the relaxation of chromatin structure [39], [40]. In contrast to SCE-I or laser induced damage, X-ray irradiation does not result in the formation of detectable ACF1 foci [39]. However we could observe ACF1 foci following charged particle irradiation in murine cells (data not shown). This observation supports the hypothesis that ACF1 plays a direct role in DSB repair. Our studies with charged particle irradiation revealed a slight reduction of DSB mobility after knockdown of ACF1 (Fig. 2B) which however was not significant.

To investigate if a mediator protein involved in a wider spectrum of repair processes upstream of the chromatin remodeler ACF1 had a more pronounced impact on DSB mobility we analyzed the effect of the radiation-induced polyADP-ribosylation of proteins and histones by poly(ADP-ribose)polymerase (PARP). PARP1 is one of the first proteins accumulating at sites of broken DNA after induction of DNA damage [41] and is involved in different repair pathways [42]–[44]. PARP1 covalently attaches poly-ADP-ribose (PAR) moieties on itself and other acceptor proteins like histones and DNA repair factors. These PAR chains are subsequently degraded by poly(ADP-ribose)glycohydrolase (PARG) and the dynamic turnover of PAR acts in a signal cascade for DNA repair, checkpoint control, apoptosis and the maintenance of genomic integrity [45]. The negative charge of poly-ADP-ribose results in an electrorepulsive interaction with DNA leading to an opening of the chromatin structure [46]. Moreover it has been shown, that active PARP1 modifies and removes histone H1 which facilitates local chromatin relaxation [47].

To test whether these polyADP-ribosylation-mediated changes in chromatin structure influence mobility of broken DNA, we inhibited PARP1 or knocked down the antagonist PARG respectively. Under the applied conditions, inhibition of PARP1 results in complete suppression of poly(ADP)ribosylation as seen after application of 20 mM H_2_O_2_, known to trigger a massive PARP1 response [27] (Fig. 2F). Knockdown of PARG reduces the endogenous protein level about 90% (Fig. 2E) and impairs degradation of PAR (Fig. S5). Msd plots (Fig. 2C,D) of both PARP inhibition or knockdown of PARG clearly show that the extent of DSB mobility is not significantly influenced by changes in polyADP-ribosylation.

### Inhibition of ATM confines mobility of broken chromatin sites

**Figure 3:**
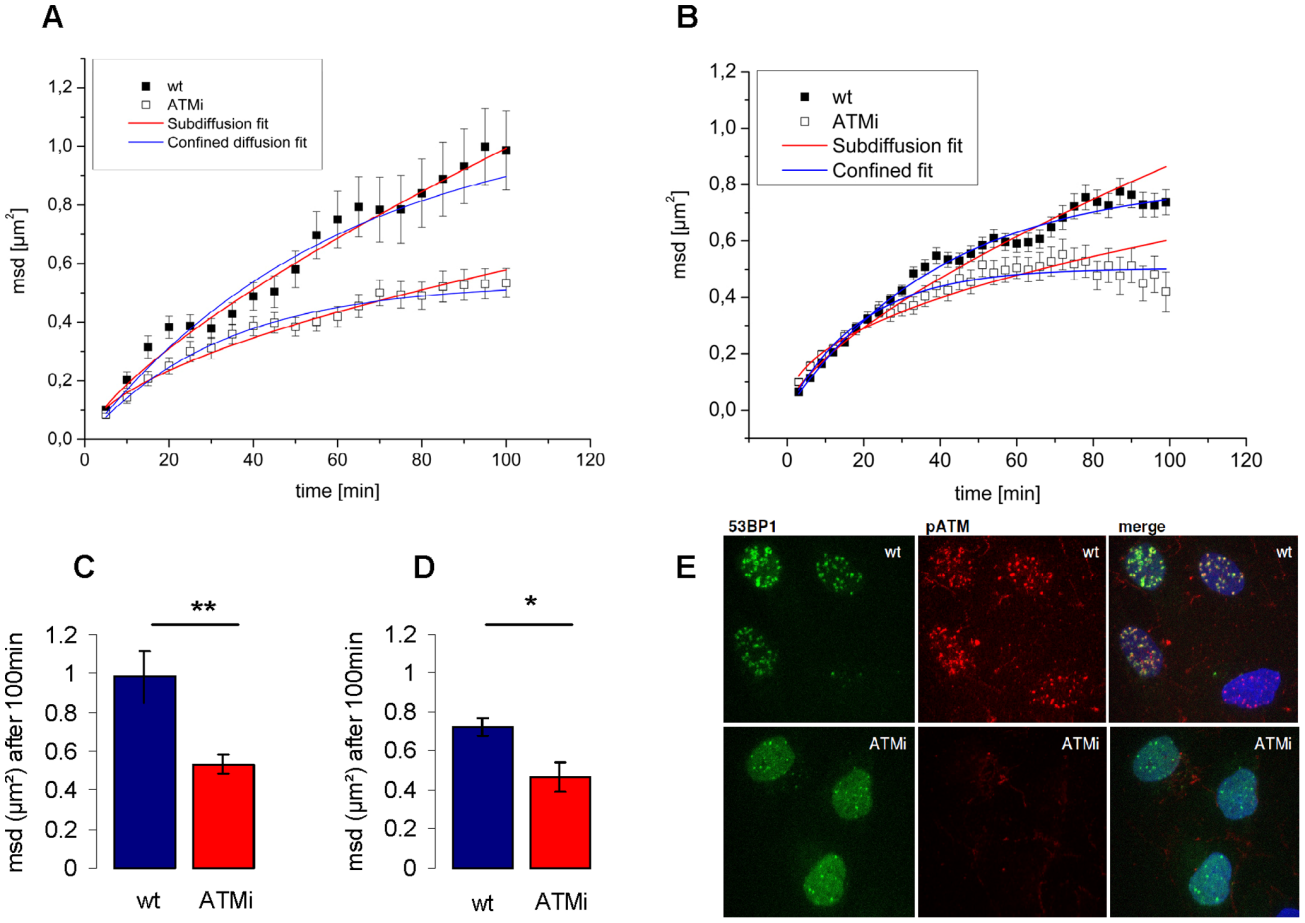
Inhibition of ATM constricts mobility of 53BP1 foci induced by heavy ion or photon irradiation. Irradiation of U2OS cells was performed by Cr (LET: 2630 keV/μm) for plots A and C and by 1 Gy X-rays for plots B and D. The mean square displacement (msd) of IRIF is plotted over time. Errors represent SEM. **A, B**) Msd plots of control (solid squares) (Cr n = 11, X-ray n = 21) and ATM inhibited cells (KU55933 open squares) (Cr n = 31, X-ray n = 11) fitted for subdiffusion (red line) and confined diffusion (blue line). **C,D**) Bar graphs of the average msd after 100 min observation time by live cell microscopy for control and ATM inhibited cells (KU55933) after irradiation with Cr **C**) and after irradiation with 1 Gy X-rays **D**). **E**) U2OS-53BP1-GFP cells irradiated with 1 Gy X-rays, fixed after 30 min and stained for pATM (red) and DNA (blue). Wt compared to cells treated with 15 μM KU55933 for 2 hours (ATMi) show efficiency of ATM kinase inhibition.

**Figure 4:**
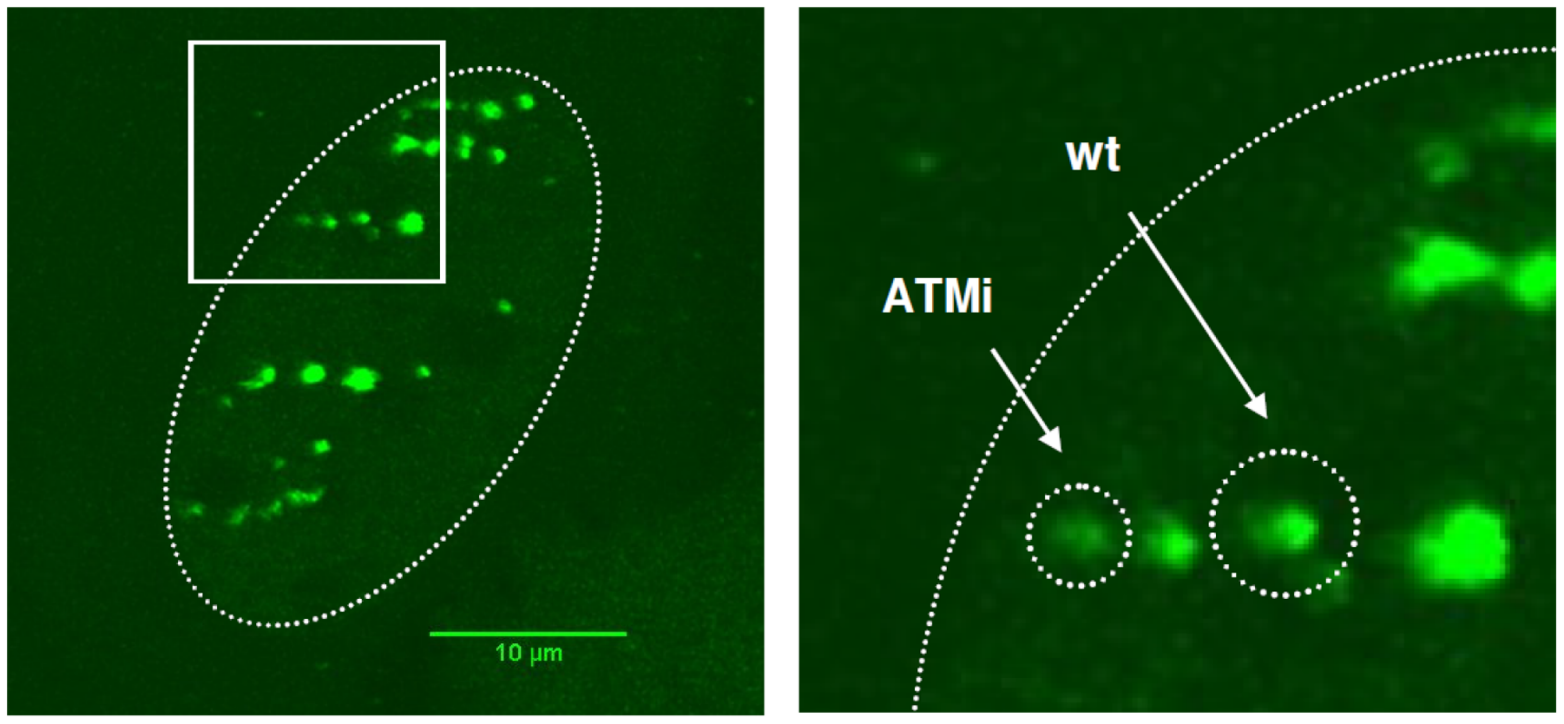
Reduced confinement radius of IRIF in U2OS cells after inhibition of ATM. A) U2OS cell stably expressing 53BP1 after low angle irradiation with C (170 keV/μm). B) Magnification of the cell nucleus. Cells and calculated confinement areas (see eq. 2) are marked by dotted lines. For simplification, both radii were exemplarily shown in the same nucleus. 3D Confinement volumes in ATM inhibited cells are decreased by a factor of 3 compared to the reference volumes of non treated cells.

Induction of DSBs activates several signal cascades to promote DNA repair. Initiated by binding of the MRE11–RAD50–NBS1 (MRN) complex, ataxia-telangiectasia mutated (ATM) kinase is recruited and activated from an inactive dimer to an active monomer by autophosphorylation of serine 1981 [48], [49]. ATM belongs to the phosphatidylinositol 3-kinase-like protein kinases (PIKK) family, which includes ATR (ataxia telangiectasia and RAD53 related) and DNA-PK_CS_ (catalytic subunit of the DNA-dependent protein kinase), which all participate in DNA damage signaling [50]. Since phosphorylation by ATM plays a key role in DSB repair we analyzed the effect of ATM activity on DSB mobility. Depending on its concentration, caffeine inhibits PIKK family proteins. We chose 10 mM caffeine primarily to inhibit ATM and to a lesser extent ATR. DNA-PKcs has a higher IC_50_ value for caffeine and should be only slightly affected by this treatment [51]. By analyzing mobility of 53BP1 foci we observed a strong bend in the msd starting about 30 min to one hour after irradiation (Fig. S1). To verify that the kinase function of ATM accounts for this effect we measured mobility of ion-induced DSB foci using the more specific inhibitor Ku55933 (Fig. 3A). Radiation induced recruitment of activated ATM to sites of DSBs is hampered after specific inhibition of ATM kinase as seen by immunoflurescence staining of pATM (Fig. 3E). A drastic reduction in the msd from 1.0 μm^2^ in control to 0.53 μm^2^ in ATM inhibited cells was found at the end of the observation period of 100 min (Fig. 3C). Using the subdiffusion model, this corresponds to a drop in the α value from 0.72±0.03 in wildtype to 0.56±0.02 in ATM inhibited cells. We conclude that inhibition of ATM provokes a strong disturbance of a random walk as seen by the reduced α value and the change of the diffusion coefficient D_c_ from 5.12_×_10^−5^ μm^2^/s for wildtype cells to 4.58_×_10^−5^ μm^2^/s. Fits for subdiffusion as well as for confined diffusion both match the experimental data with a similar R^2^ (R^2^ = 0.98 for subdiffusion vs. 0.99 for confined mobility in ATM inhibited samples and R^2^ = 0.98 for subdiffusion vs. 0.96 for confined mobility in wildtype cells). Using the model of confined motion, the predicted confinement radius for DSB mobility in wildtype cells was 1.05±0.07 μm while ATM inhibition resulted in confinement to a much smaller radius of 0.73±0.01 μm in the corresponding experiment (for comparison see Fig. 4). This reduction of the confinement radius leads to a reduction of the sampled volume within the cell nucleus by a factor of 3 (4.85 μm^3^ vs 1.63 μm^3^). To examine if this effect arises from the induction of high numbers of DSBs in close proximity by heavy ion irradiation, which was shown to lead to a hyperactivation of ATM [52], we repeated the experiments with 1 Gy of photon irradiation (X-rays), thus inducing isolated DSBs. Msd plots confirmed a confinement in mobility due to inhibition of ATM also in the case of photon irradiation (Fig 3B,D). Taken together the experiments show a significant decrease in mobility due to inhibition of ATM, independent of irradiation quality. Different ion species with different LET values ranging from C (170 keV/μm) to U (15000 keV/μm) were used in our experiments. We have no indication for a direct LET effect on mobility (data not shown). This is consistent with the previously described msd values for nickel ions (LET: 3430 keV/μm) and low LET photons (X-rays) which are in the same range [28]. During this study, we did notice some variability in absolute values of movement kinetics in individual experiments in heavy ion as well as in photon irradiated samples. This could be attributed to influences of the cell lot and cultivation time used, as the experiments were executed over a longer time period. To keep the influence of this variability on the obtained results as low as possible we carried out repeated measurements in independent experiments for each condition and directly compared only results of contiguous experiments.

### Missing concatenation of DNA strands by suppression of cohesin or MRN complex formation does not influence DSB mobility

**Figure 5:**
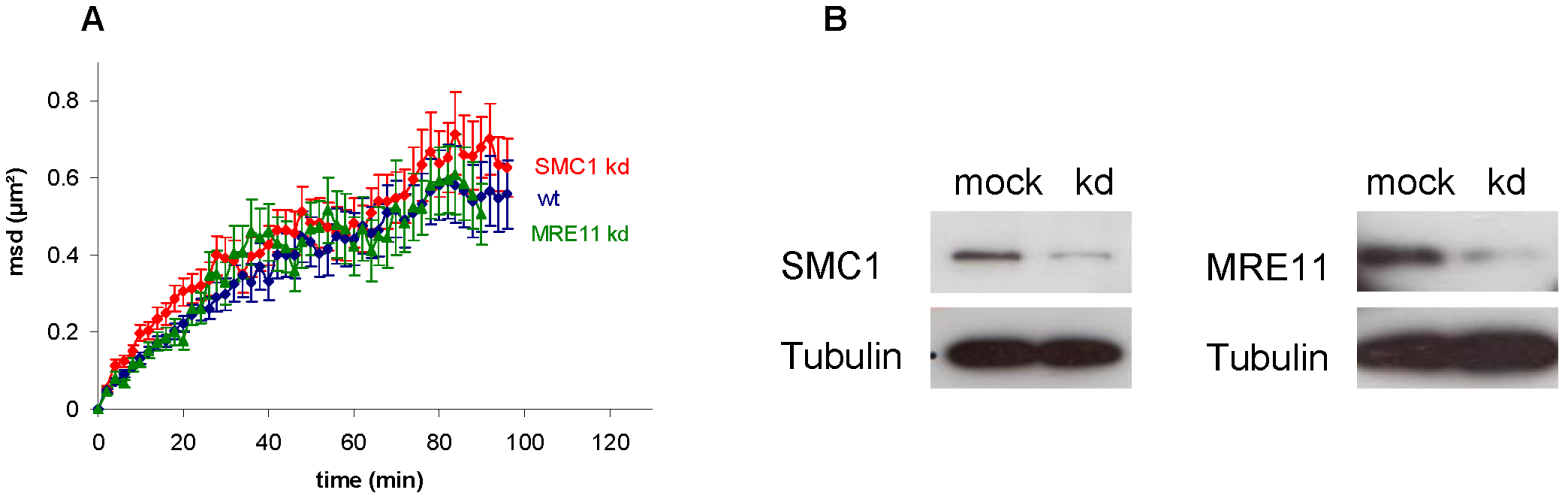
Knockdown of SMC1 or MRE11 does not influence mobility of IRIF. A) Mean square displacement (msd) of 53BP1 foci after irradiation with Pb (LET: 13500 keV/μm) is plotted against time for wt (blue line), SMC1 knockdown (red line) and MRE11 knockdown cells (green line). B) Western blots of U2OS cells 48 h after knockdown of SMC1 and MRE11 with tubulin as loading control.

Not only a change in chromatin density surrounding the DSB, but also a direct interaction of DNA strands could account for differences in mobility kinetics. A reduction of mobility might arise from tethering broken and non-broken DNA strands. We chose to study two protein complexes which are proposed to stabilize DNA strands at sites of DSBs [53], [54]: the cohesin complex, which maintains sister chromatid cohesion and helps strand invasion in HR repair [55], and the MRN complex that is known to tether broken DNA strands [56]. The MRN complex binds to DSBs and is often considered to be responsible for holding broken ends in close proximity to facilitate repair [57], [58]. In this context, two RAD50 and two MRE11 proteins act together as an ATP-driven molecular clamp [59], [60]. According to this model, the loss of the tethering complex would locally decrease the stability of chromatin, especially in the vicinity of multiple DSBs as expected to be induced by heavy ion irradiation. To test this hypothesis we knocked down MRE11 and analyzed whether mobility of IRIF is enhanced or not (Fig. 5B). The reduced MRE11 level is sufficient to prevent normal accumulation of MRE11 to DSBs as seen after irradiation with X-rays (Fig. S6). Surprisingly, no significant differences between the mobility of wildtype and MRE11 knockdown cells were observed (Fig. 5A). Obviously, the accumulation of the MRN complex at the break site did not influence the DSB mobility on the tested temporal and spatial scale.

In contrast to the MRN complex which is considered to tether the DNA strands only in the vicinity of DSBs, the cohesin complex generally keeps sister chromatids in close proximity, a process which might facilitate homology search in DNA repair by HR [54], [61]. This cohesion might also reduce the mobility of DSBs in G2/S. Since the heterodimer SMC1/SMC3 forms a major subunit of the cohesin complex and loss of SMC1 would prevent the complex formation, we addressed the DSB mobility under conditions of SMC1 knockdown (Fig. 5B). Inhibiting the cohesion complex formation by knockdown of SMC1 did not influence the dynamic behavior of DSB sites as measured by our approach (Fig. 5A). Similarly, the knockdown of the human SCC2 analogue loading factor NIPBL to prevent loading of the functional cohesin complex to DNA, showed no altered movement characteristic compared to wildtype or SMC1 depleted cells (Fig. S2). The findings that neither MRE11 nor cohesin influences movement of DSBs suggests that not the connection of broken DNA strands but more likely changes in the surrounding chromatin structure largely define the dynamic behavior of DSBs on the time scale of minutes to hours.

## Discussion

Mobility of both DSBs and undamaged chromatin has been described in yeast and in mammalian cells using a variety of approaches. In the context of chromatin organization, movement in combination with proximity of induced DSBs is considered to contribute to the formation of chromosome rearrangements [62], [63] which can favor genomic instability. Recently, Roukos et al. showed by separate labeling of both DNA ends that break ends pair before undergoing translocations [63]. Indeed, a higher probability for chromosomal translocations between proximal chromosome territories in interphase could be demonstrated [64], [65], thus connecting the roaming of damaged chromatin to the volume of potentially wrong interaction partners. Recent studies in budding yeast relate enhanced mobility following DSB induction to a facilitated homology search for HR, which is the predominant repair pathway in this eukaryote [9], [66]. In agreement with other studies we show that motion properties and diffusion coefficients are generally lower in mammals than those observed in yeast [67]. The reduced mobility of DSBs in mammals could probably be connected to the use of NHEJ as the major pathway independent of the cell cycle [54], [68]. The average mobility of IRIF of untreated cells in our study is in a range similar to observations by others in mammalian cells [15], [18], [63]. However absolute values might also depend on the time-regime and methods of evaluation used in different studies, e.g. comparison of the relative motion of neighboring spots yielded generally smaller absolute values for the diffusion of DSBs [11]. Given that mammalian nuclei have a far larger volume than yeast nuclei, foci in human cells can explore a much smaller percentage of the nuclear volume and chromatin is expected to be the restrictive factor for confinement in diffusion. In mammalian cells DSBs are produced within chromatin and thus their motion characteristics come along with the general mobility of chromatin. Compaction of chromatin seems to be one factor affecting mobility as heterochromatic areas were shown to induce a relocation of DSBs to euchromatic areas at the periphery of heterochromatin [19], [69]. This relocation is accompanied by a local DNA decondensation along the particle trajectory after ion irradiation [19], [20] or in the vicinity of DSB after γ-rays [14] raising the question whether local decondensation supports mobility of DSBs.

Various enzymes like PARP, ACF1 and other chromatin remodeler complexes can modulate the structure of chromatin, inducing a local decondensation in response to DNA damage [37]. The importance of chromatin organization is demonstrated by the higher mutation levels found in heterochromatic compared to euchromatic regions in cancer genomes [70] and by a reduced repair efficiency in heterochromatic areas [4], [71].

Since an increased mobility of free ends may trigger the formation of chromosomal aberrations, we wanted to investigate the extent to which nuclear components, known to influence chromatin structure after ionizing radiation, might impact the movement of damaged DNA. Considering that the absence of the repair mediator 53BP1 was shown to reduce mobility of uncapped telomeres [17] we first verified by comparison with U2OS NBS1-GFP cells that the mobility of IRIF is not influenced by overexpression of 53BP1 (Fig. S3). The strong reduction of IRIF mobility after partial ATP depletion (Fig. 2) demonstrated the validity of our setup and analysis method and confirmed the strong energy dependency of chromatin structure and DSB mobility shown previously [15], [18], [72]. We suspected the drastic reduction of mobility after ATP depletion to be at least partly related to chromatin remodeling processes in the proximity of DSBs. To test this hypothesis we downregulated the chromatin remodeler ACF1 by siRNA treatment. ACF1 plays a direct role in DSB repair [39], [73], possibly by remodeling chromatin structure in the surrounding of DSBs. The slight reduction in the slope of the msd curve was not significantly below the wt (Fig. 2). We conclude that silencing of this remodeler alone only marginally affects the mobility of IRIF in the spatial scale addressed by our system. As multiple chromatin remodeler complexes are involved in chromatin rearrangements during DNA repair [74], maybe working partially redundant, it is likely that knockdown of a single remodeler does not affect the chromatin structure to a larger extent.

In this context we expected polyADPribosylation by PARP to have a stronger effect on chromatin structure as it not only transiently links proteins and histones but further recruits remodeling enzymes. This recruitment either leads to a transient repressive chromatin structure followed by subsequent opening mediated by the degradation of PARylation by PARG, [74] or directly generates an open, accessible state [75]–[77]. Mechanisms by which polyADPribosylation provokes a local relaxation of chromatin structure in the environment of DSBs include the electrostatic repulsion of chromatin by the negative charges of polyADPribose as well as destabilized nucleosomal interactions with DNA [46]. Inhibition of PARP as well as stabilization of polyADPribosylation by knockdown or PARG surprisingly showed no significant influence on DSB mobility (Fig. 2). This suggests that the resulting changes in chromatin structure by the radiation-induced PARylation either have only minor effects on the compaction or act only very locally or temporally restricted and thus do not influence a longer range mobility of DNA lesions. A similar explanation might apply to the absence of a measurable increase in DSB mobility by the knockdown of cohesin or MRE11. MRE11 tethers DNA strands by forming a complex with RAD50 [56], which we expected to increase the positional stability around DNA ends. However, it is not known if this affects only the mobility of the two break ends on a small scale or also the surrounding chromatin which is probed in our assay. A different approach in which individual ends of a single DSB can be visualized [13] would be needed to clarify this question. Cohesin on the other hand is not specifically acting at damaged DNA sites but is more generally needed for sister chromatid cohesion. However it is also recruited to break sites and facilitates DNA repair during HR by tethering chromatids [55]. In our human U2OS cells neither knockdown of the cohesion core component SMC1 nor the cohesion loading factor NIPBL [78] resulted in pronounced changes of chromatin dynamics (Fig. 5 and Fig. S2). We have to conclude that cohesion has no effect on movement characteristics of mammalian IRIF as measured by our approach. Given that cohesion of sister chromatids during HR takes place only in S/G2 phase of the cell cycle and might also be necessary for checkpoint activation [61], [79], diminishing the cohesin complex should exert effects primarily on cells in S or G2 phase. In yeast, which utilized HR throughout the cell cycle, it was recently shown that cohesin indeed constrains the mobility of RAD52 foci [80]. The cell cycle distribution in our experiments was determined to be around 50% G1 cells by flow cytometry (Fig. S4). In our analyses we were not able to distinguish two populations of distinct movement kinetics as expected if knockdown of cohesin had a strong effect only in G2 phase. However, future live cell experiments using additional expression of a cell cycle marker might help clarify the contribution of chromatin modifying factors on DSB mobility in different cell cycle phases.

Induction of DSBs creates a local decondensation at least in heterochromatic areas, which is clearly detectably after charged particle irradiation [19], [20]. It is expected that decondensation facilitates repair by increasing the accessibility of break ends for repair factors [19], [81]. In heterochromatic areas ATM is involved in the local decondensation after induction of DSBs through phosphorylation of KAP1 and a fraction of long persisting DSBs associated with heterochromatic regions arise after inhibition of ATM, both after X-rays as well as carbon ion irradiation [4], [19], [71]. Though ATM is a central player in many aspects of the DNA damage response affecting γ-H2AX formation as well as MDC1 recruitment, 53BP1 IRIF still form after inhibition of ATM activity. Remarkably we observed a significant reduction in mobility of IRIF after inhibition of ATM as indicated by the more pronounced bending downwards of the msd curves (Fig. 3). We hypothesize is that this effect is caused by a change in chromatin condensation state. The observation that the msd curve shows a distinct confinement following ATM kinase inhibition suggests a mechanism of chromatin relaxation using its enzymatic activity. Our observation that ATM activation is needed for reduced confinement is in line with an increased mobility of DSB break sites compared to non-damaged chromatin [18]. However, as the phosphorylation substrates of ATM act in cell-cycle arrest, apoptosis and DNA repair [82] it will be difficult to pinpoint the effectors responsible for changes in chromatin and DNA mobility. Krawczyk et al. described a decreased mobility of heterochromatic breaks 24 h after induction of DSBs [18]. Even though similar to our experiments inhibition of ATM led to a reduced mobility different mechanisms ought to apply. At early time points 53BP1 foci are considered to belong mainly to euchromatic areas which comprise around 80% of the chromatin. In addition it was shown that 53BP1 can only penetrate into heterochromatic areas after their decondensation [20], [62]. At later time points (24 h), persisting foci as observed by Krawczyk et al. [18] are expected to be mainly associated to heterochromatic areas due to slower repair kinetics [4], [71].

In contrast to heterochromatin, it is still unknown if and how ATM is involved in the decondensation processes of euchromatin. It is possible that some opening of the chromatin structure around the breaks is also necessary in euchromatic areas for efficient DSB repair. A missing local relaxation of chromatin would lead to confined mobility of broken DNA due to the physical constraints of the chromatin surrounding DNA breaks. Previous studies using different irradiation qualities suggested that either early DSB repair factors or physical forces could be responsible for a local repair-associated decondensation [19], [62].

Overall our findings prove that mobility of DSB containing chromatin domains is not influenced by small scale chromatin remodeling. Even tethering of the damaged DNA strands which facilitates correct joining of DSBs did not significantly influence mobility of the surrounding chromatin domains. The confinement of mobility due to ATM inhibition provides new hints into the regulation of local chromatin relaxation. Furthermore it may open up new opportunities to investigate the interplay between IRIF dynamics and the formation of chromosomal translocations.

## Supporting Information

Figure S1 **Inhibition of ATM by caffeein or KU55933 reduces the msd of radiation induced 53BP1 foci.** Mean square displacement (msd) of 53BP1 foci after irradiation with C (170 keV/μm) is plotted against time for wt (blue line) and cells inhibited by 10 mM caffeine (green line). For comparison the msd plot of ATM inhibited cells (15 μM KU55933) irradiated with Cr (2630 keV/μm) is shown (red line). Specific ATM inhibition as well as inhibition by caffeine reduces mobility of 53BP1 foci.
(TIFF)

Figure S2 **Knockdown of NIPBL does not alter the msd of radiation induced 53BP1 foci.** Mean square displacement (msd) of 53BP1 foci after irradiation with U (15000 keV/μm) is plotted against time for wt (blue line) and NIPBL knockdown cells (green line). B) Western blots of U2OS cells 48 h after knockdown of NIPBL.
(TIFF)

Figure S3 **Comparison of mobility of NBS1 and 53BP1 foci.** Msd of U2OS cells stably expressing NBS1-GFP or 53BP1-GFP tracked over one hour after irradiation with X-rays (1 Gy) show no differences in foci mobility.
(TIF)

Figure S4 **FACS analyses of cell cycle distribution in U2OS-53BP1-GFP cells.** Cell cycle distribution was measured for GFP positive cells and revealed 46% G1 cells and 26% G2 cells.
(TIFF)

Figure S5 **Functional validation of PARG knockdown efficiency.** USOS cells 48 h after mock treatment (left) or knockdown of PARG (right) treated with 1 mM H_2_O_2_ for 5 min (upper row) and subsequent incubation in culturing media for 20 minutes (lower row). H_2_O_2_ induces PARP mediated poly(ADP)ribosylation (green). The degradation of PAR (green) is diminished in cells knocked down of PARG.
(TIF)

Figure S6 **Functional validation of MRE11 knockdown efficiency.** U2OS cells 48 h after mock treatment (upper row) or knockdown of MRE11 (lower row) irradiated with 2 Gy X-rays and fixed after 15 minutes incubation. While γH2AX foci (red) still form, Mre11 (green) recruitment is strongly hampered in MRE11 downregulated cells. DNA is stained in blue by Dapi.
(TIF)

Table S1 Sequences of siRNAs used in our experiments.
(DOC)
