# Supplementary figures and images for: ATM Alters the Otherwise Robust Chromatin Mobility at Sites of DNA Double-Strand Breaks (DSBs) in Human Cells
Source: PLoS One. 2014 Mar 20;9(3):e92640. doi: 10.1371/journal.pone.0092640 (PMC3961414; doi:10.1371/journal.pone.0092640)

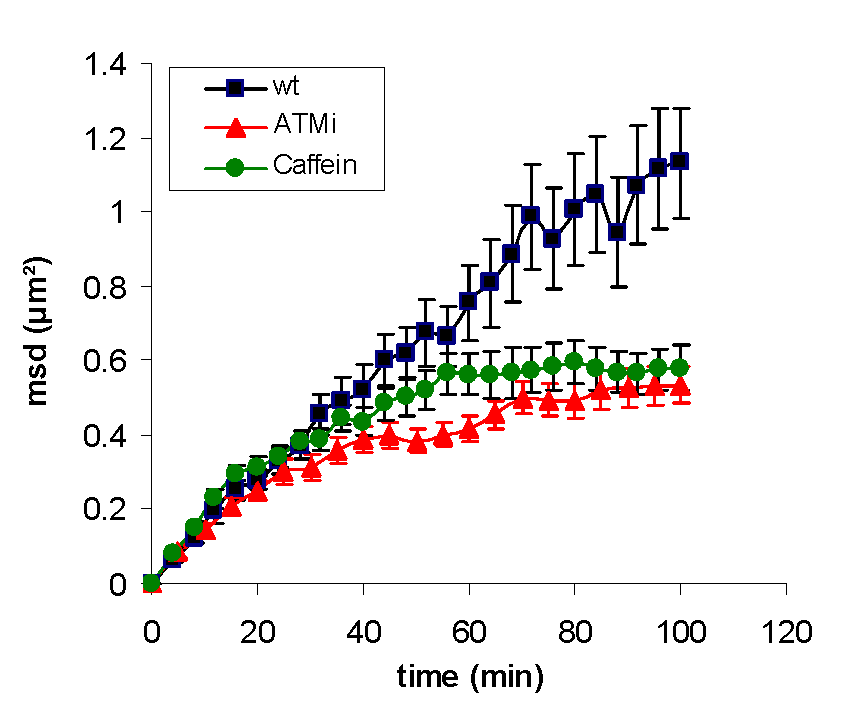

Supplement: Figure S1 — Inhibition of ATM by caffeein or KU55933 reduces the msd of radiation induced 53BP1 foci. Mean square displacement (msd) of 53BP1 foci after irradiation with C (170 keV/μm) is plotted against time for wt (blue line) and cells inhibited by 10 mM caffeine (green line). For comparison the msd plot of ATM inhibited cells (15 μM KU55933) irradiated with Cr (2630 keV/μm) is shown (red line). Specific ATM inhibition as well as inhibition by caffeine reduces mobility of 53BP1 foci. (TIFF) [file pone.0092640.s001.tif]

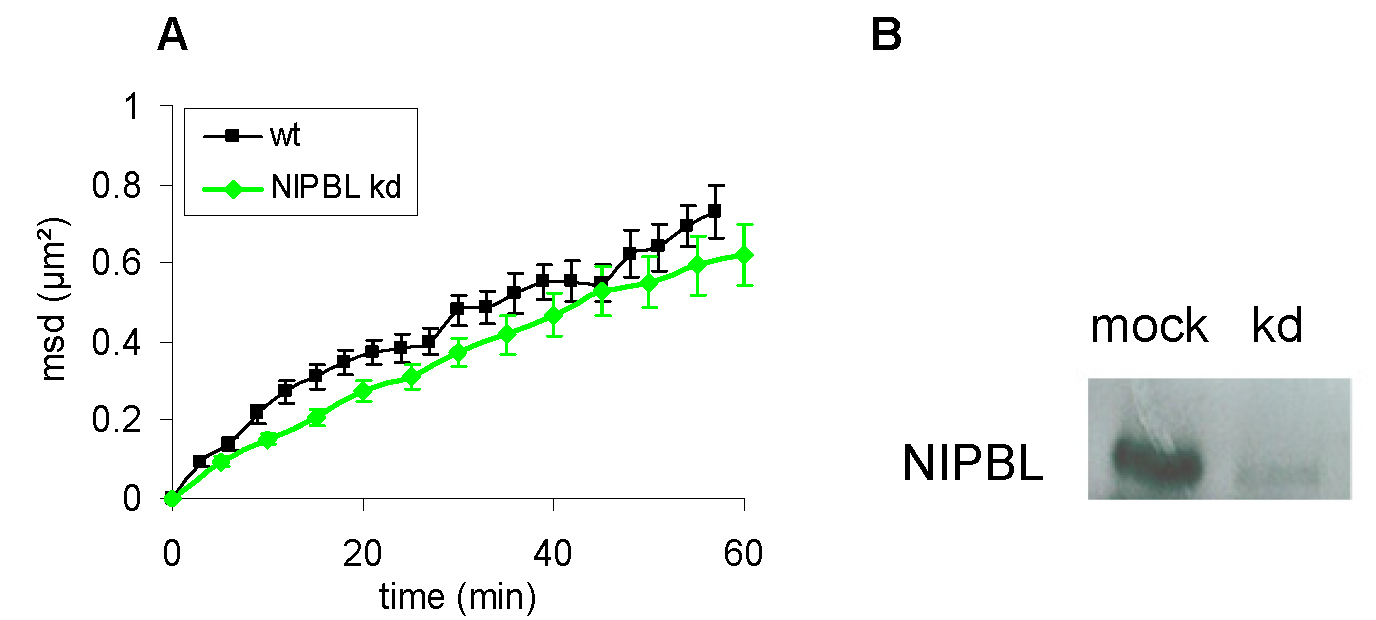

Supplement: Figure S2 — Knockdown of NIPBL does not alter the msd of radiation induced 53BP1 foci. Mean square displacement (msd) of 53BP1 foci after irradiation with U (15000 keV/μm) is plotted against time for wt (blue line) and NIPBL knockdown cells (green line). B) Western blots of U2OS cells 48 h after knockdown of NIPBL. (TIFF) [file pone.0092640.s002.tif]

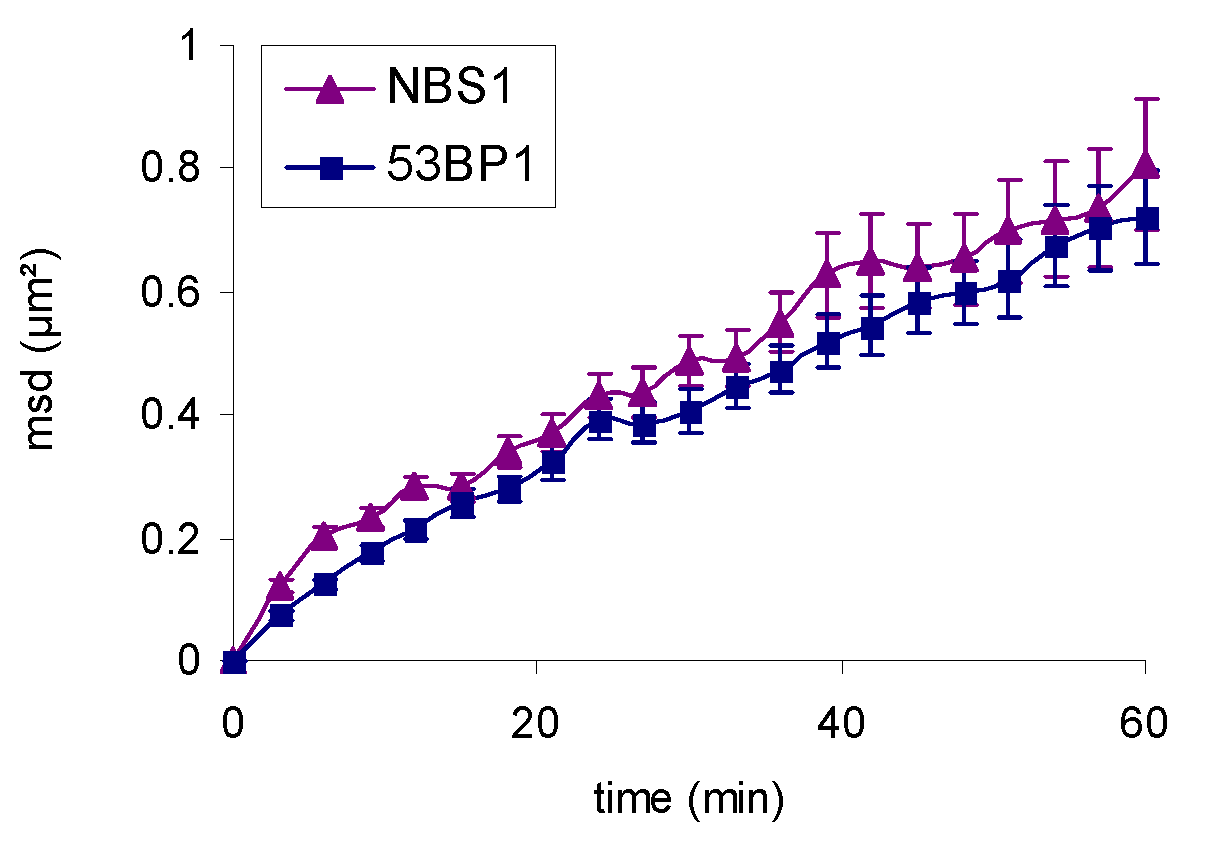

Supplement: Figure S3 — Comparison of mobility of NBS1 and 53BP1 foci. Msd of U2OS cells stably expressing NBS1-GFP or 53BP1-GFP tracked over one hour after irradiation with X-rays (1 Gy) show no differences in foci mobility. (TIF) [file pone.0092640.s003.tif]

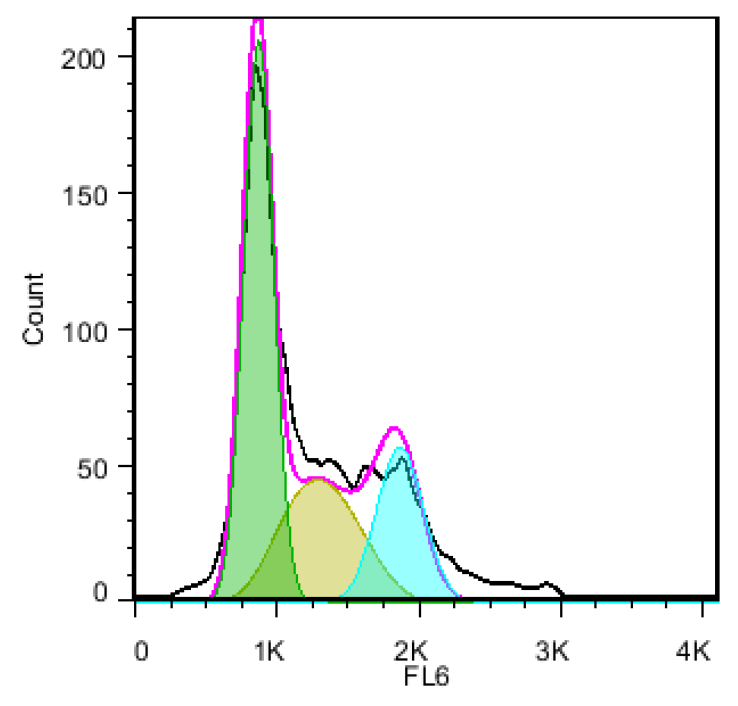

Supplement: Figure S4 — FACS analyses of cell cycle distribution in U2OS-53BP1-GFP cells. Cell cycle distribution was measured for GFP positive cells and revealed 46% G1 cells and 26% G2 cells. (TIFF) [file pone.0092640.s004.tif]

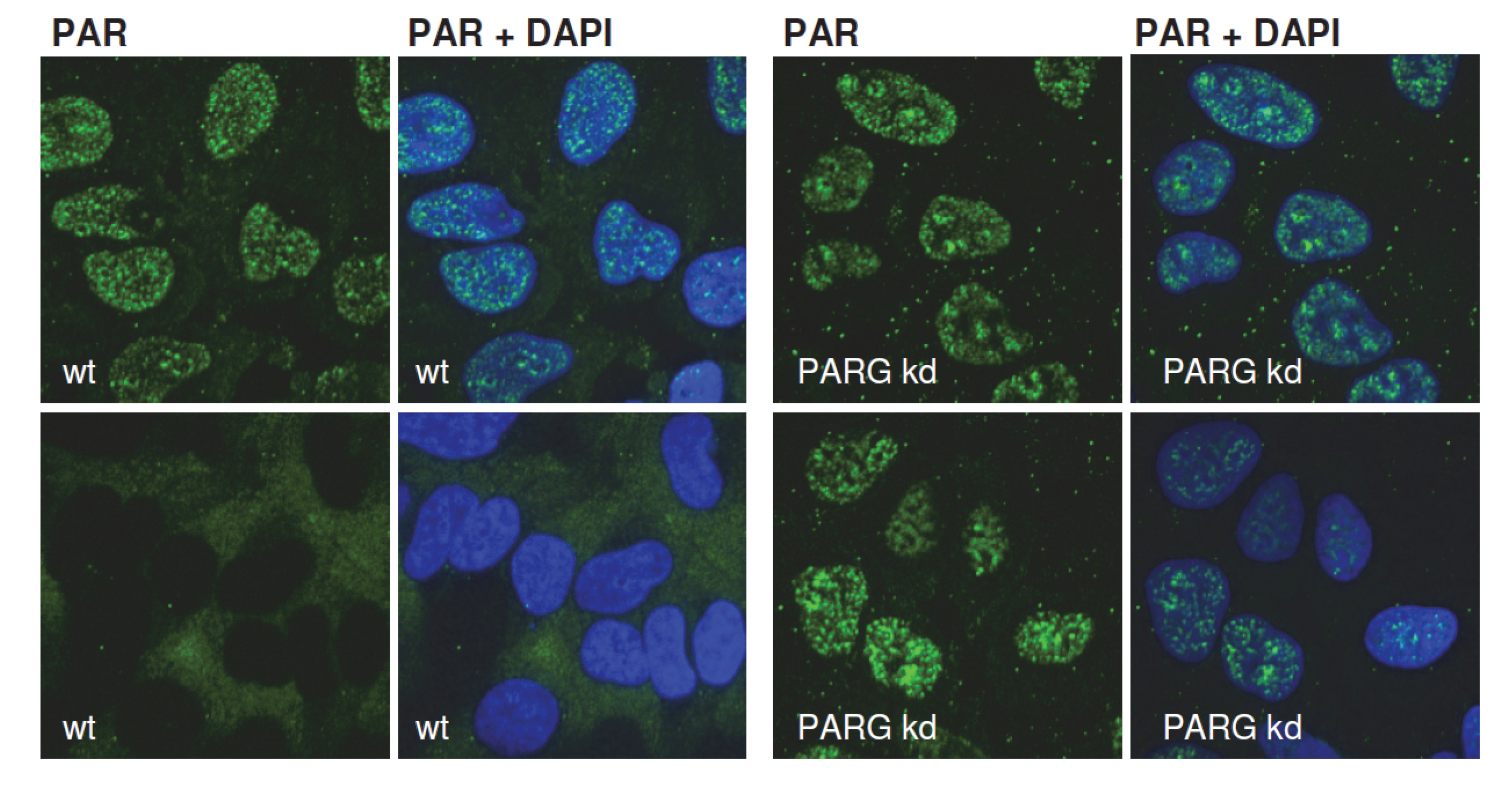

Supplement: Figure S5 — Functional validation of PARG knockdown efficiency. USOS cells 48 h after mock treatment (left) or knockdown of PARG (right) treated with 1 mM H2O2 for 5 min (upper row) and subsequent incubation in culturing media for 20 minutes (lower row). H2O2 induces PARP mediated poly(ADP)ribosylation (green). The degradation of PAR (green) is diminished in cells knocked down of PARG. (TIF) [file pone.0092640.s005.tif]

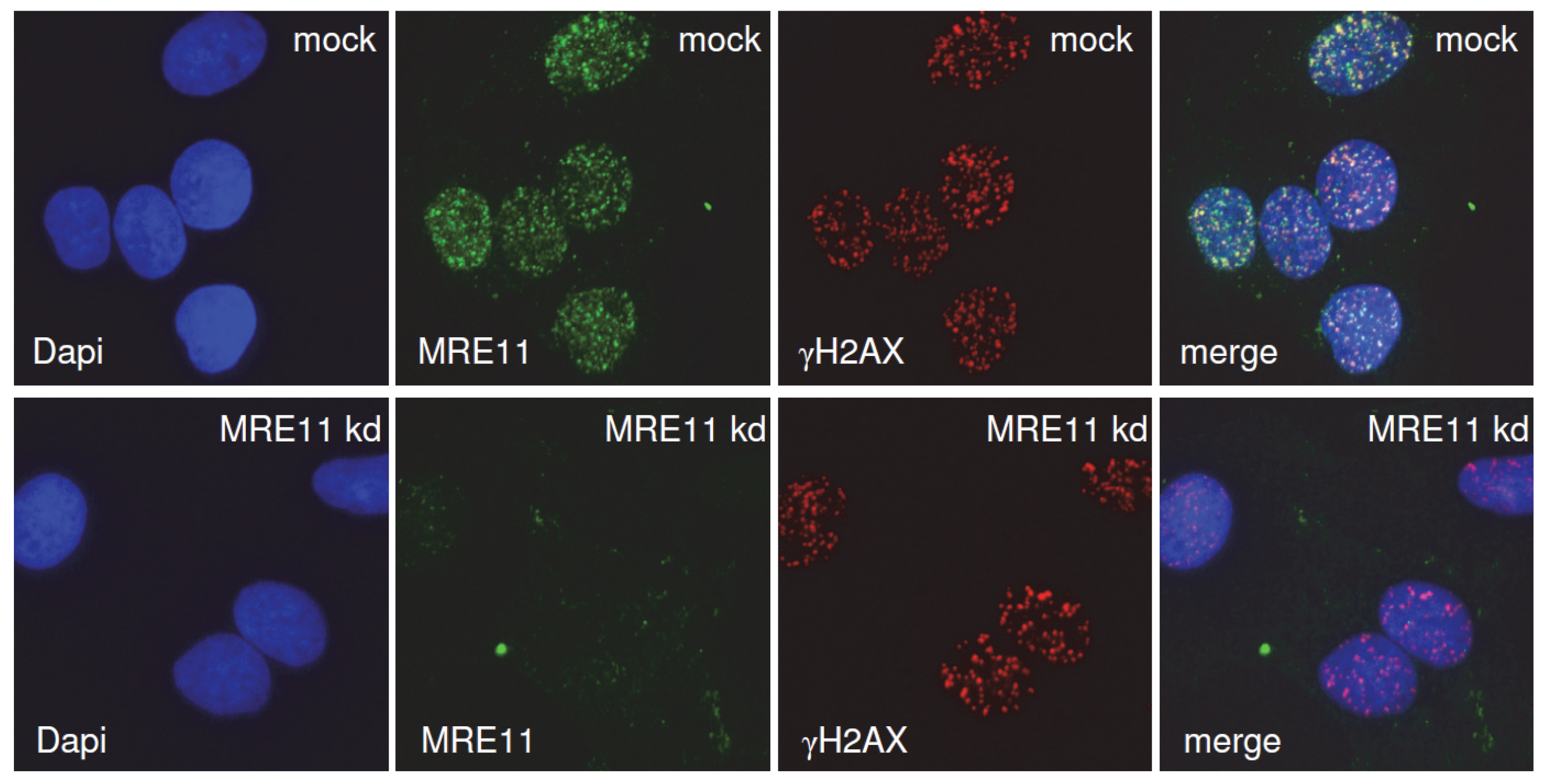

Supplement: Figure S6 — Functional validation of MRE11 knockdown efficiency. U2OS cells 48 h after mock treatment (upper row) or knockdown of MRE11 (lower row) irradiated with 2 Gy X-rays and fixed after 15 minutes incubation. While γH2AX foci (red) still form, Mre11 (green) recruitment is strongly hampered in MRE11 downregulated cells. DNA is stained in blue by Dapi. (TIF) [file pone.0092640.s006.tif]
