# Supplementary material for: ATM Alters the Otherwise Robust Chromatin Mobility at Sites of DNA Double-Strand Breaks (DSBs) in Human Cells
Source: PLoS One. 2014 Mar 20;9(3):e92640. doi: 10.1371/journal.pone.0092640 (PMC3961414; doi:10.1371/journal.pone.0092640)
Supplement: Table S1 — Sequences of siRNAs used in our experiments. (DOC) [file pone.0092640.s007.doc]

| **Protein** | **siRNA sequence** |
| --- | --- |
| PARG | AAATGGGACTTTACAGCTTTG |
| NIPBL | CAAAAGAAGCAGAAGAAAA |
| SMC1 | GCAAUGCCCUUGUCUGUGA |
| ACF1 | AACACUGUGAAGCACAAGAUG |
| MRE11 | GGAGGUACGUCGUUUCAGA |
